# Role of fried foods and oral/pharyngeal and oesophageal cancers

**DOI:** 10.1038/sj.bjc.6602542

**Published:** 2005-04-26

**Authors:** C Galeone, C Pelucchi, R Talamini, F Levi, C Bosetti, E Negri, S Franceschi, C La Vecchia

**Affiliations:** 1 Istituto di Ricerche Farmacologiche ‘Mario Negri’, Via Eritrea 62, 20157 Milan, Italy; 2 Servizio di Epidemiologia e Biostatistica, Centro di Riferimento Oncologico, Via Pedemontana Occ.le, 33081 Aviano (Pordenone), Italy; 3 Registre vaudois des tumeurs, Institut universitarie de médicine sociale et préventive, CHUV-Falaises 1, 1011 Lausanne, Switzerland; 4 International Agency for Research on Cancer, 150 COURS Albert Thomas, F-69372 Lyon, cedex 08, France; 5 Istituto di Statistica Medica e Biometria, Università degli Studi di Milano, Via Venezian 1, 20133 Milan, Italy

**Keywords:** oral neoplasms, pharyngeal neoplasms, oesophageal neoplasms, fried foods, risk factors, case–control study

## Abstract

We investigated the role of fried foods on oral-pharyngeal and oesophageal cancers, using data from two case–control studies conducted in Italy and Switzerland between 1992 and 1999, one with a total of 749 (634 men) cases of oral/pharyngeal cancer and 1772 (1252 men) controls, the other with 395 (351 men) cases of oesophageal cancer and 1066 (875 men) controls. Controls were admitted for acute, non-neoplastic conditions, unrelated to alcohol and smoking consumption. After allowance for sex, age, centre, education, body mass index, tobacco smoking, alcohol drinking and nonalcohol energy intake, the multivariate odds ratios (ORs) for an increment of one portion per week of total fried foods were 1.11 (95% confidence interval (CI): 1.05–1.17) for oral-pharyngeal and 1.16 (95% CI: 1.08–1.26) for oesophageal cancer. The ORs were consistent across strata of gender (OR in men only were 1.10 and 1.16, respectively), age, alcohol, tobacco consumption and body mass index.

Cancers of the oral cavity/pharynx and oesophagus are two of the most common cancers in the world, and their incidence and mortality rates are higher in Southern Europe than in the rest of Europe (La Vecchia *et al*, 2004; Levi *et al*, 2004). These neoplasms are strongly related to smoking and alcohol consumption (Franceschi *et al*, 1990; Negri *et al*, 1993), but several studies have related various aspects of diet to the risk of oral/pharyngeal and oesophageal cancers (Trichopoulou *et al*, 2000; Zheng *et al*, 1992). A diet rich in animal fats has been associated with an increased risk of these cancers (Launoy *et al*, 1998), but the epidemiological evidence on the role of meat consumption is not consistent (Franceschi *et al*, 1999b). A number of studies reported inverse associations with consumption of different types of meat (Launoy *et al*, 1998), while several other studies showed an increased risk, mainly related to barbecued and fried meat (Yu *et al*, 1988; De Stefani *et al*, 1990), suggesting that the cooking method could be involved in upper digestive tract carcinogenesis. The guidelines on diet of the American Cancer Society reported that high-temperature cooking methods (as in frying) give rise to a wide variety of mutagenic substances that are carcinogenic in experimental animals (Weinhouse *et al*, 1991).

Several studies have reported a direct relation between fried meat and cancers of the colon, rectum and stomach (IARC, 1993; Navarro *et al*, 2004). Other studies found a positive association between fried foods and laryngeal (Bosetti *et al*, 2002), breast (Dai *et al*, 2002), lung (Sinha *et al*, 1998), pancreatic (Anderson *et al*, 2002) and lower urinary tract (Steineck *et al*, 1990) cancers.

A Brazilian case–control study showed that habitual intake of bacon and fried foods was a risk factor for oral cancer (Toporcov *et al*, 2004). Pharyngeal cancer was also associated with a high consumption of meat and fried foods in a case–control study in Spain (Escribano Uzcudun *et al*, 2002).

Concerning oesophageal cancer, some studies (Yu *et al*, 1988; De Stefani *et al*, 1990) showed an increased risk, but others (Ward *et al*, 1997) found no association with frequent fried beef consumption.

We investigated the issue of fried meat and other fried foods using data from a large, multicentric case–control study.

## MATERIALS AND METHODS

Data were obtained from two coordinated hospital-based case–control studies with the same design, questionnaire and inclusion criteria. In brief, the first study (Levi *et al*, 2000; Soler *et al*, 2001) was conducted between 1991 and 1997 in Italy (Pordenone, Udine, Rome, Latina) and in Switzerland and included 749 (634 men) cases of cancer of the oral cavity and pharynx (median age 57 years) and 1772 (1252 men) controls (median age 57 years) matched on age, sex and study centre. The second study (Soler *et al*, 2001) was conducted between 1992 and 1999 in Italy (Milan, Pordenone, Padua, Udine) and Switzerland and included 395 (351 men) cases of cancer of the oesophagus (median age 60 years) and 1066 (875 men) controls (median age 60 years) matched on age, sex and study centre. All cancer cases were incident and histologically confirmed. Controls were patients hospitalized for a wide spectrum of acute non-neoplastic conditions (overall, 23% had nonalcohol-related traumas, mostly fractures and sprains, 26% nontraumatic orthopedic disorders, 30% acute surgical conditions and 21% miscellaneous other illnesses such as eye, ear or skin diseases).

Cases and controls were aged ⩽79 years and were identified and questioned by trained interviewers during their hospital stay, in the same network of teaching and general hospitals in the areas under surveillance. The proportion of refusals was less than 5% in cases and controls in both studies.

Since oral-pharyngeal and oesophageal cancers are more frequent in men, and to avoid heterogeneity between sexes of the covariates included in the model, the major analyses were restricted to men and the results on female subjects were given in the text only.

The same structured questionnaire was used in both studies, including information on sociodemographic factors, anthropometric variables, smoking, alcohol and other lifestyle habits, a problem-oriented medical history, physical activity and history of cancer in relatives. Information on diet referred to the previous 2 years and was based on a validated and reproducible food frequency questionnaire (FFQ) (Franceschi *et al*, 1993; Decarli *et al*, 1996), comprising 78 foods, food groups or recipes, and allowing the estimation of total energy intake. The FFQ was divided into six section: (1) bread, cereals, first courses; (2) second courses (i.e., meat, fish and other main dishes); (3) side dishes (i.e., vegetables, fried/baked potatoes); (4) fruits; (5) sweets, desserts and soft drinks; (6) milk, hot beverages and sweeteners. At the end of each section, one or two open questions were used to include foods that were not in the questionnaire, but were eaten at least once per week. Among the items in the FFQ, some questions referred specifically to consumption of fried foods, as beef, fish, eggs and omelettes, and potatoes. It was asked for the weekly frequency of consumption of fried foods, as well as for their usual portion size. No information was obtained on the common degree of browning of fried foods.

Odds ratios (ORs), and their corresponding 95% confidence intervals (CIs), for an increment of one portion per week of fried foods, were computed using conditional multiple logistic regression. All regression models were matched on age and study centre, and included terms for education (<7, 7–<12, ⩾12 years), body mass index (in quintiles), alcohol consumption (in quintiles: for oral/pharyngeal cancer, cut points were 10, 22.5, 37.75, 61 glasses per week, and for oesophageal cancer were 8, 22.5, 35.5, 59 glasses per week, plus a term for ex-drinkers), smoking habits (never, ex, current smokers of <15, 15–<25, ⩾25 cigarettes per day) and nonalcohol energy intake (in quintiles). Population attributable risks (PAR) were derived from multivariate ORs, using the method described by Bruzzi *et al* (1985) and Mezzetti *et al* (1996).

## RESULTS

Table 1 shows various demographic, lifestyle and dietary characteristics among 634 male cases of oral-pharyngeal cancer and 1252 controls, and 351 male cases of oesophageal cancer and 875 controls. In both studies, cases smoked tobacco and drank alcohol much more frequently than controls. Current smokers were 71.7% of oral-pharyngeal cancer cases and 32.3% of corresponding controls, and 59.5% of oesophageal cancer cases and 32.0% of corresponding controls located. In the highest quintile of alcohol consumption were 46.6% of oral cancer cases and 8.3% of controls, and 47.0% of cases of oesophageal cancer and 10.3% of corresponding controls. Total consumption of fried foods of cases was higher than controls: the average number of portions per week of cases was 3.0 portions in both studies, as compared to 2.54 and 2.65 for controls.

In Table 2, cases and controls of the two cancers studied are distributed by level of intake of various types of fried foods. Linear trends in risk, adjusted for several potential confounding factors, assessing the difference in the distributions of the indicated types of fried foods between cases and controls, are also shown. Cases reported higher intakes of fried beef, eggs and total foods, and increasing trends in risk.

Table 3 reports the ORs of oral-pharyngeal and oesophageal cancers according to different levels of intake of total fried foods, after adjustment for several potential confounding factors. The ORs of oral-pharyngeal cancer were 1.24 and 1.22 for an intake of 1–<2 and ⩾2 portions of fried foods per week respectively, and the corresponding ORs for oesophageal cancer were 1.32 and 1.43. The OR for an increment of one portion of fried foods per week was 1.10 (95% CI: 1.04–1.17) for oral-pharyngeal cancer and 1.16 (95% CI: 1.07–1.25) for oesophageal cancer. When we computed risks in women using the same regression model, the ORs for total fried foods for female subjects were substantially similar to those in men (continuous OR=1.23, 95% CI: 1.06–1.42 for oral and pharyngeal cancers and OR=1.14, 95% CI: 0.83–1.55 for oesophageal cancer), and for men and women combined the ORs were 1.11 and 1.16, respectively.

In Table 4 are considered the continuous ORs according to total fried foods consumption, in separate strata of age, alcohol drinking, tobacco smoking and body mass index. No significant heterogeneity emerged across any of the strata considered (*P*-values not shown).

## DISCUSSION

This study found that a diet rich in fried foods was related to a moderate increase in risk of upper aero-digestive tract cancers in men. These findings follow a recent companion study from Bosetti *et al* (2002), which found that consumption of fried foods was directly associated to laryngeal cancer risk. The observed risk may be explained by a direct action of carcinogens produced by frying, and is plausible since there is a direct combination between these substances and the upper respiratory tract. In fact, during the frying of protein-rich food, such as meat and fish, genotoxic heterocyclic amines (HCAs) are formed (Pfau and Marquardt, 2001). The most important variables contributing to the formation of HCAs are: cooking temperature (>150°C), cooking time (>2 min) and cooking method (frying, oven grilling/broiling, barbecuing) (Thomson, 1999). Heterocyclic amine compounds formed in the cooking of certain foods have been shown to be bacterial mutagens and animal carcinogens. In rats, these amines induce cancer specifically in sites such as breast, colon or pancreas, which are associated with Western-type diet, where promotional elements such as dietary fat play an enhancing role (Weisburger and Jones, 1990).

However, other factors, besides HCA, could give explanation to the positive association observed between fried foods and these neoplasms. Some studies found that a diet rich in meat and eggs, independently from the cooking methods, lead to an increased risk of oral and oesophageal cancers (Franceschi *et al*, 1999a; Bosetti *et al*, 2000). Also, a possible explanation of the increased risks may be linked to the fat used for frying. In fact, in Italy, eggs, which presented the strongest direct associations with cancer risk in both of these studies, and beef are frequently fried using butter, whose detrimental effect on upper aero-digestive tract cancers was found in several studies (Launoy *et al*, 1998; Franceschi *et al*, 1999a; Bosetti *et al*, 2002). However, fish and potatoes are usually fried in oils, mostly olive oil and specific or mixed seeds oil, and it was found that these types of oils were protective for oral/pharyngeal and esophageal cancers (Franceschi *et al*, 1999a; Bosetti *et al*, 2000).

In order to reduce potential information bias, in this study the questionnaire was administered to both cases and controls by the same interviewers, under similar conditions, and controls were selected among patients with admission diagnosis not related to tobacco smoking, alcohol drinking and diet modifications. Bias in the recall of food intake should be limited in this not particularly health conscious population, and given the comparability between cases and controls in hospital-based settings (D'Avanzo *et al*, 1997). The high participation rate of cases and controls, the comparable catchment areas of study subjects, the careful adjustment for tobacco and alcohol, as well as for other potential confounding factors, are among the strengths of the study. Moreover, the use of a substantially reproducible and valid questionnaire (Franceschi *et al*, 1993; Decarli *et al*, 1996) allowed to take into account the full dietary pattern and to adjust for total energy intake (Willett and Stampfer, 1986; Franceschi *et al*, 1999b).

Nevertheless, the increase in risk was moderate and bias or confounding may account at least for part of it. Fried food consumption may simply be an indicator of a generally less healthy lifestyle (Utter *et al*, 2003), although we carefully controlled in the regression models for education and/or lifestyle covariates including tobacco and alcohol consumption. If the associations are real, the adjusted PARs for fried foods in men would be 14% (95% CI: 0–37.9%) for oral/pharyngeal cancers and 21% (95% CI: 0–47.6%) for esophageal cancer, further confirming the importance of diet in the aetiology of these neoplasms (Negri *et al*, 1993).

## Figures and Tables

**Table 1:** Basic demographic, life-style and dietary characteristics among 634 male cases of oral/pharyngeal cancer and 1252 male controls, and 351 male cases of oesophageal cancer and 875 male controls, Italy and Switzerland, 1992–1999

	**Oral cavity/pharynx**		**Oesophagus**	
	**Cases^a^**	**Controls^a^**	**Cases^a^**	**Controls^a^**
Age (years)	56.9 (8.9)	56.6 (10.8)	60.0 (8.2)	59.2 (9.0)
Education (years)	7.3 (3.7)	8.0 (3.9)	7.2 (3.6)	8.5 (4.1)
Body mass index (kg m^−2^)	24.45 (3.7)	26.28 (3.3)	25.32 (3.5)	26.26 (3.3)
Nonalcohol energy intake (kcal day^−1^)	2370 (735)	2449 (807)	2354 (882)	2378 (787)
				
*Tobacco smoking*				
Never smokers	20 (3.1)	346 (27.6)	22 (6.3)	244 (27.9)
Ex-smokers	160 (25.2)	502 (40.1)	120 (34.2)	351 (40.1)
Current smokers (cigarettes per day)	454 (71.7)	404 (32.3)	209 (59.5)	280 (32.0)
<15	72 (11.4)	132 (10.5)	35 (10.0)	93 (10.6)
15–<25	230 (36.3)	186 (14.9)	100 (28.5)	119 (13.6)
⩾25	152 (24.0)	86 (6.9)	74 (21.0)	68 (7.8)
				
*Alcohol consumption (quintile of intake)* b				
1st	31 (5.8)	310 (26.0)	5 (1.6)	213 (25.5)
2nd	37 (6.9)	305 (25.5)	22 (7.0)	217 (26.0)
3th	76 (14.2)	279 (23.4)	52 (16.6)	177 (21.2)
4th	142 (26.5)	200 (16.8)	87 (27.8)	142 (17.0)
5th	250 (46.6)	100 (8.3)	147 (47.0)	85 (10.3)
Ex-drinkers	98 (15.5)	58 (4.6)	38 (10.8)	41 (4.7)
				
*Fried food consumption (portions per week)*				
Beef/veel	0.37 (0.4)	0.33 (0.4)	0.38 (0.4)	0.31 (0.4)
Fish/shellfish	0.39 (0.4)	0.36 (0.4)	0.44 (0.5)	0.42 (0.5)
Eggs/omelettes	1.34 (2.0)	0.99 (1.3)	1.24 (1.4)	1.05 (1.4)
Potatoes	0.92 (0.8)	0.79 (0.8)	0.87 (1.4)	0.79 (0.8)
Total fried foods	3.02 (2.8)	2.54 (2.0)	2.96 (2.5)	2.65 (2.2)

a For the quantitative variables, we present the mean value and in parentheses the standard errors, while for categorical variables we present absolute numbers and percentages in parentheses.

b Cut points for oral/pharyngeal cancer were 10, 22.5, 37.75, 61 glasses per week, and for oesophageal cancer 8, 22.5, 35.5, 59 glasses per week.

**Table 2:** Distribution of 634 male cases and 1252 male controls of oral/pharyngeal cancer, and 351 male cases and 875 male controls of oesophageal cancer, according to fried food consumption, Italy and Switzerland, 1992–1999

	**Level of intake**			
**Fried food (servings per week)**	**1 (lowest)**	**2**	**3 (highest)**	***Z*^a^,^b^ for trend**
	Oral cavity/pharynx			
*Beef/veal* c				
Cases	365	169	100	0.65
Controls	739	336	177	
				
*Fish/shellfish* c				
Cases	311	223	100	1.26
Controls	677	358	217	
				
*Eggs/omelettes* c				
Cases	186	120	328	2.70
Controls	449	268	535	
				
*Potatoes* c				
Cases	184	151	299	0.01
Controls	390	312	550	
				
*Total fried foods* d				
Cases	96	143	395	0.90
Controls	231	294	727	
				
	Oesophagus			
*Beef/ veal* c				
Cases	184	127	40	1.94
Controls	535	235	105	
				
*Fish/shellfish* c				
Cases	166	133	52	−0.20
Controls	429	281	165	
				
*Eggs/omelettes* c				
Cases	88	107	156	2.69
Controls	276	234	365	
				
*Potatoes* c				
Cases	121	109	121	−0.14
Controls	283	236	356	
				
*Total fried foods* d				
Cases	60	94	197	1.49
Controls	175	212	488	

a Estimates from conditional logistic regression conditioned on age, centre, and adjusted for education, body mass index, tobacco smoking, alcohol drinking and nonalcohol energy intake.

b *Z* values are interpretable as standard normal deviates.

c The first level of intake was less then 0.5 portion per week, the second level was 0.5–<1portion per week, the third level was ⩾1 portion per week.

d The first level of intake was less then 1 portion per week, the second level was 1–<2 portions per week, the third level was ⩾2 portions per week.

**Table 3:** Odds ratios (OR) and corresponding 95% confidence intervals (CIs) according to total fried food consumption among 634 male cases and 1252 male controls of oral/pharyngeal cancer, and 351 male cases and 875 male controls of oesophageal cancer, Italy and Switzerland, 1992–1999

	**Level of intake (portions per week)**			
**Total fried foods**	**<1**	**1–<2**	**⩾2**	**Continuous OR^c^**
*Oral cavity/pharynx*				
OR^a^ (95% CI)	1^b^	1.24 (0.83–1.85)	1.22 (0.85–1.74)	1.10 (1.04–1.17)
				
*Oesophagus*				
OR^a^ (95% CI)	1^b^	1.32 (0.83–2.12)	1.43 (0.92–2.24)	1.16 (1.07–1.25)

a Estimates from conditional logistic regression conditioned on age, centre, and adjusted for education, body mass index, tobacco smoking, alcohol drinking and nonalcohol energy intake.

b Reference category.

c For an increment of one portion per week.

**Table 4:** Odds ratios (ORs) and corresponding 95% confidence intervals (CIs) according to fried food consumption in strata of selected covariates among 634 male cases and 1252 male controls of oral/pharyngeal cancer, and 351 male cases and 875 male controls of oesophageal cancer, Italy and Switzerland, 1992–1999

	**OR^a^ (95% CI)**							
	**Age (years)**		**Alcohol (drinks week^−1^)**		**Tobacco smoking**		**Body mass index (kg m^−2^)**	
	**<60**	**⩾60**	**<Median value^b^**	**⩾Median value**	**Non-smokers^c^**	**Smokers^d^**	**<26**	**⩾26**
*Oral cavity/pharynx*								
Total fried foods	1.13	1.08	1.02	1.16	1.00	1.13	1.12	1.09
	(1.03–1.23)	(0.99–1.18)	(0.92–1.14)	(1.06–1.26)	(0.83–1.20)	(1.06–1.22)	(1.03–1.23)	(1.00–1.19)
								
*Oesophagus*								
Total fried foods	1.24	1.13	1.14	1.17	1.16	1.17	1.14	1.19
	(1.09–1.42)	(1.02–1.27)	(0.98–1.32)	(1.06–1.30)	(0.95–1.43)	(1.07–1.27)	(1.01–1.28)	(1.06–1.34)

a For an increase of one serving per week; estimates from conditional logistic regression conditioned on age and study centre, and adjusted for education, body mass index, tobacco smoking, alcohol drinking and nonalcohol energy intake.

b Median value for oral/pharyngeal cancer was 31 glasses per week, for oesophageal cancer 29.75 glasses per week.

c Including never smokers and ex-smokers since 20 or more years.

d Including smokers and ex-smokers since less than 20 years.
